# Representation of selected-reaction monitoring data in the mzQuantML data standard

**DOI:** 10.1002/pmic.201400281

**Published:** 2015-06-05

**Authors:** Da Qi, Craig Lawless, Johan Teleman, Fredrik Levander, Stephen W Holman, Simon Hubbard, Andrew R Jones

**Affiliations:** 1 Institute of Integrative Biology, University of LiverpoolLiverpool, UK; 2 The Faculty of Life Sciences, University of ManchesterManchester, UK; 3 Department of Immunotechnology, Lund UniversityLund, Sweden; 4 Bioinformatics Infrastructure for Life Sciences (BILS), Lund UniversityLund, Sweden

**Keywords:** Bioinformatics, mzQuantML, Proteomics standards initiative, SRM

## Abstract

The mzQuantML data standard was designed to capture the output of quantitative software in proteomics, to support submissions to public repositories, development of visualization software and pipeline/modular approaches. The standard is designed around a common core that can be extended to support particular types of technique through the release of semantic rules that are checked by validation software. The first release of mzQuantML supported four quantitative proteomics techniques via four sets of semantic rules: (i) intensity-based (MS^1^) label free, (ii) MS^1^ label-based (such as SILAC or N^15^), (iii) MS^2^ tag-based (iTRAQ or tandem mass tags), and (iv) spectral counting. We present an update to mzQuantML for supporting SRM techniques. The update includes representing the quantitative measurements, and associated meta-data, for SRM transitions, the mechanism for inferring peptide-level or protein-level quantitative values, and support for both label-based or label-free SRM protocols, through the creation of semantic rules and controlled vocabulary terms. We have updated the specification document for mzQuantML (version 1.0.1) and the mzQuantML validator to ensure that consistent files are produced by different exporters. We also report the capabilities for production of mzQuantML files from popular SRM software packages, such as Skyline and Anubis.

SRM is a selective analysis strategy using MS, and is typically employed in quantitative proteomics experiments [1,2]. SRM is gaining popularity for detection and quantification of target peptides, selected as surrogates for the quantification of their parent proteins assuming a 1:1 stoichiometry, with high accuracy and precision across multiple samples in a highly multiplexed manner. The SRM approach is typically performed on triple quadrupole (QqQ) mass spectrometers, exploiting the ability of the two quadrupole mass analysers to act as selective *m*/*z* filters. The instrument is operated in a targeted manner, whereby the first quadrupole (Q1) is set to transmit the *m*/*z* value of the precursor ion of a peptide of interest. The ionized peptide then enters a collision cell filled with neutral gas, whereupon fragmentation takes place, causing the peptide ion to dissociate into several product ions. One of these product ions is then directed through the third quadrupole (Q3) for detection, again by selecting a specific *m*/*z* value for transmission. The precursor-product ion pair is referred to as a transition, and in most cases several transitions are monitored serially for a given peptide before the detection of a different peptide commences. The area (or on occasion height) of the chromatographic peak generated through monitoring one or several transitions for a given peptide is then used for quantification purposes. The selection of transitions for peptides of interest is crucial to the design of an SRM experiment, and hence metadata related to them should be captured. In addition, the quantitative values (chromatographic peak area or height) produced by processing software should be recorded to allow further use of the data.

The HUPO proteomics standards initiative (PSI) has been developing data standards and minimum reporting guidelines for proteomics for over ten years—these include standards such as mzML for raw data or peak lists [3], mzIdentML for identification data [4], mzQuantML for quantitation data [5] and mzTab—as a simple summary of the overall results (http://www.psidev.info/mztab). The TraML standard [6] has been developed to capture the transitions that are *input* to the process, but to date, no PSI standard has been able to capture the *output* of quantitative software for SRM data. In terms of public databases for proteomics, international efforts are being coordinated via the ProteomeXchange consortium [7], under which umbrella the PRIDE [8] database accepts submissions for “discovery” or global analyses, and the PASSEL repository accepts quantitative SRM datasets [9].

When mzQuantML was initially released [5], the specifications contained four sets of “semantic rules” checked by the validation software [10] to support (i) intensity-based (MS^1^) label free, (ii) MS^1^ label-based (such as SILAC or N^15^), (iii) MS^2^ tag-based (iTRAQ or tandem mass tags), and (iv) spectral counting. The core data structures in an mzQuantML file are *features—*defined as a quantified (often 2D) region of LC-MS (MS^1^) space, peptides, proteins, and protein groups. For these core data types, quantification data can be stored in mzQuantML in flexible 2D matrix structures called *QuantLayers*. A second orthogonal set of concepts are defined in mzQuantML as *Assay* (one sample measured by MS), *StudyVariable* (e.g. a collection of replicate *Assays* over which averaging of quantitative values may be done) and *Ratios* (in which the numerator and denominator are pairs of *Assays* or pairs of *StudyVariables*). A typical peptide-level *AssayQuantLayer* thus has rows of data about peptides that have been measured, and one column of data for each *Assay*. In the intensity-based label-free case, one *Assay* is typically mapped to one LC-MS run.

SRM data present a different set of challenges to the other four techniques supported in mzQuantML version 1.0. The primary difference is that data and meta-data about “features” in both the MS^1^ (precursors) and MS^2^ (products) domain are required, in addition to suitable descriptors to ensure that both label-free and label-based SRMs protocols can be consistently captured, as both types of protocol are widely used. Several software packages have been developed specifically for SRM data analysis, including Skyline [11] and Anubis [12]. Our primary focus was to ensure that data formats produced from these packages (and others) can be converted to a valid mzQuantML file, maintaining all the essential data and metadata, such that it can be read by other data processing or visualization packages, and uploaded to public repositories.

The mzQuantML schema contains an <*AnalysisSummary*> element to store information about the technique producing data encoded in the file. It is a compulsory element in mzQuantML, allowing any postprocessing software package to determine the technique used, and what data types to expect in the file. One specific controlled vocabulary (CV) term for the SRM technique (i.e. “SRM quantitation analysis”) has been added to PSI-MS CV [13] for this purpose. The experimental design may also be in label-based or label-free mode, and a second term (i.e. “MS1 label-based analysis” or “LC-MS label-free quantitation analysis”) is thus also required.

One of the basic data types in mzQuantML is <*Feature*>, which is defined to mean a one or 2D feature in the MS^1^ domain. As such, a corresponding mechanism was required for SRM data such that the <*Feature*> element also could capture the essential attributes of the MS^2^ data. For this part, we have been able to reuse CV terms from the PSI-MS that are used in TraML to capture the required data at the MS^2^-level, thus leaving the <*Feature*> element and attributes to capture details about the MS^1^ data as in all other techniques. The differences and an example are shown in Table 1.

**Table 1:** The MS^1^ (precursor) and MS^2^ (product) features of an SRM experiment represented in mzQuantML

	MS^1^ (attribute of <*Feature>)*	MS^2^ (<*cvParam*> of <*Feature*>)
Retention time	Rt	Local retention time
*m/z*	*m/z*	Isolation window target *m/z*
Charge	Charge	Charge state
Other measurements (optional)	N/A	Isolation window lower offset
		Isolation window upper offset or CV terms allowed by CV rules

Example (A measured transition captured in mzQuantML.):	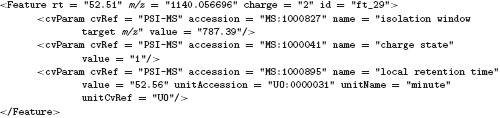	

The attributes “rt,” “mz,” and “charge” of the feature are defined to mean the retention time, mass/charge, and charge of the precursor ion, while the three CV terms (i.e. “local retention time,” “isolation window target *m/z*,” and “charge state”) capture the equivalent data for the attendant transition product (MS^2^ feature) that is quantified by the software. If the retention time of the precursor (MS^1^) is considered to be unknown by the software (although in practice the retention time of the precursor and product should be the same), a “null” value can be used.

The actual SRM “raw” data is quantifications of transition measurements (XICs), which should be represented using the <*FeatureQuantLayer*> structure (inside the <*FeatureList*>), as shown in Fig. 1. The <*FeatureQuantLayer*> is able to capture multiple different data types in the column definitions, unlike other types of *QuantLayer* (e.g. <*AssayQuantLayer*>, <*RatioQuantLayer*>, and <*StudyVariableQuantLayer*>) used for peptides, proteins or protein groups that can have only a single data type in one *QuantLayer*. In Fig. 1, four different data types produced by the Skyline software are described using CV terms. The <*DataMatrix*> then stores the actual values as arrays of values. Each row references to a <*Feature*> (as shown in Table 1) and each set of values must match the number of columns above (and this is checked by the validation software).

**Figure 1:**
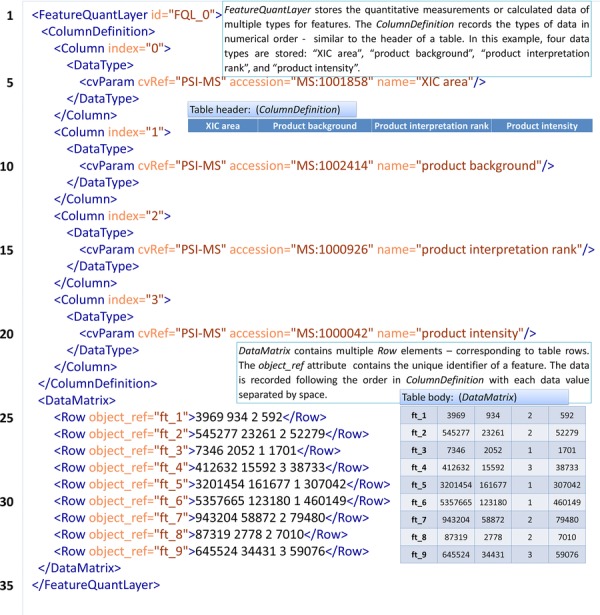
A measured transition captured in mzQuantML.

SRM quantification software also takes a mean, sum or weighted mean of different quantified transitions for the same peptide, to produce a peptide-level quantification value, and software may also average or aggregate different peptide values to produce protein-level values. Figure 2 shows how peptide-level quantification values can be captured in mzQuantML with references to all the features from which the quantitative values have been derived.

**Figure 2:**
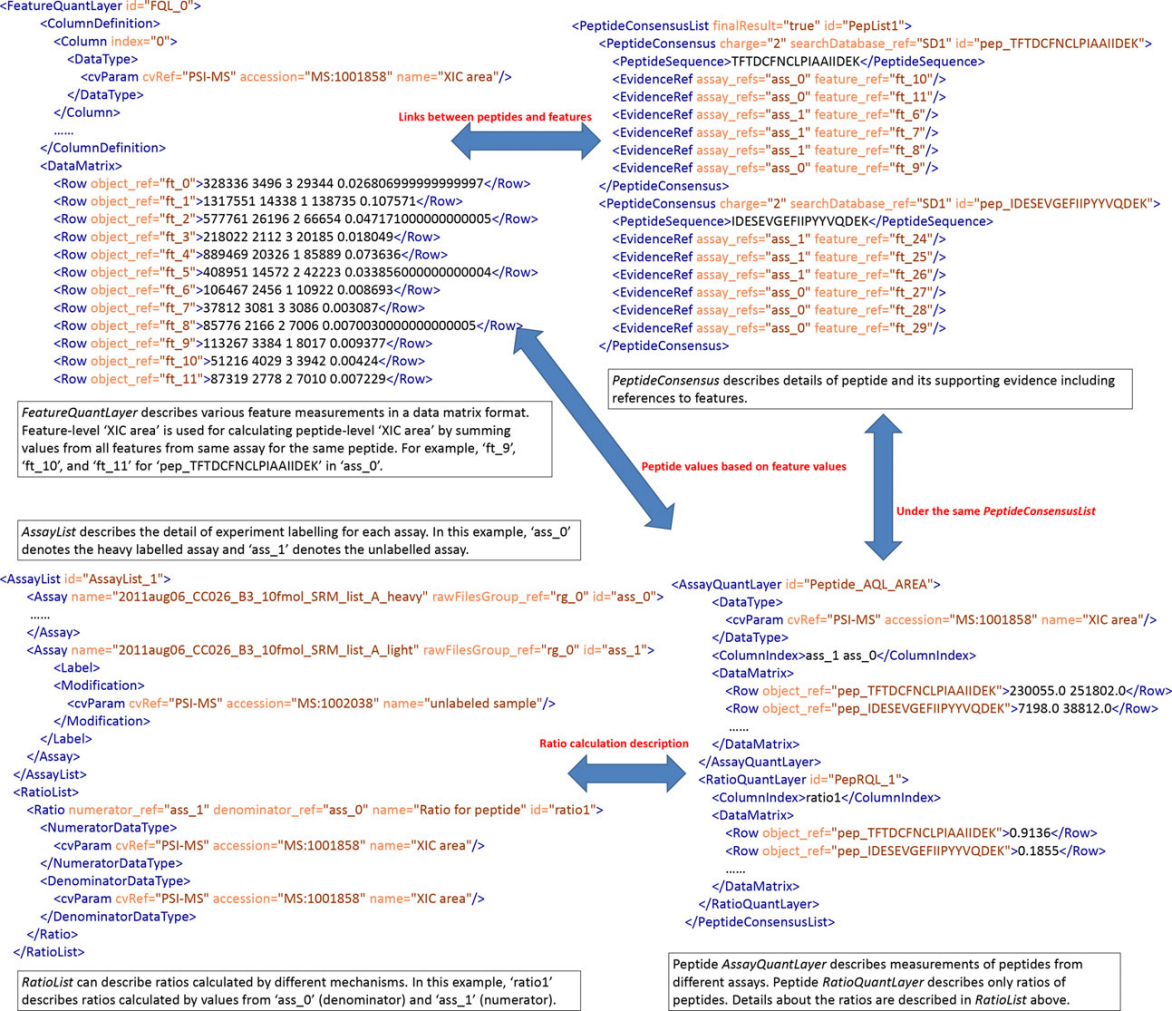
Peptide-level ratios derived from multiple transitions in a labeled SRM experiment, in which “light” and “heavy” isotope labels were used, represented in mzQuantML.

We have previously developed an mzQuantML validator [10] for supporting mzQuantML schema version 1.0.0. It checks whether mzQuantML files are syntactically or semantically valid, and contain correct CV terms in each element. Work has been done to extend the functions of the validator (https://code.google.com/p/mzquantml-validator/) for supporting SRM in mzQuantML (now at version 1.0), as follows. The validator detects that it is processing an SRM-derived file from the <*AnalysisSummary*> element and whether the file is of type LC-MS label-free or MS^1^ label-based technique also from the <*AnalysisSummary*>. The validator checks the SRM mzQuantML file against both SRM-specific rules and the general rules for the other techniques (since SRM can be viewed as a special case of label-free or label-based). It means that more rules apply to an mzQuantML file for SRM than for the other four supported quantitation techniques. The validator also checks the files against specific CV mapping rules, which contain extended rules based on general CV mapping rules. For example, <*Feature*> must contain a <*cvParam*> “isolation window target *m/z*” to capture the measured *m/z* of the targeted product ion. Otherwise, the file is not a valid SRM mzQuantML file.

One of the most popular tools for performing SRM data analysis is Skyline [11]. As such, it was deemed high priority for testing the mzQuantML encoding, and for getting community acceptance of the format, that we developed a convertor for Skyline to mzQuantML. The convertor supports files from Skyline version 1.4 and 2.X (tested on both version 1.4 and 2.6.0.6851). First, a specific “report type” in Skyline, which creates a CSV (comma separated values) formatted file with defined column types, was agreed on. A Java application that reads the special Skyline report CSV, and converts it to mzQuantML using the rules and encoding discussed above was developed. The convertor has a basic graphical user interface so that it can be used by bench scientists who do not wish to use command line tools, although a command line option is also available. It can be downloaded on http://code.google.com/p/srm-mzquantml-convertor/, along with the Skyline report type, which must be imported into Skyline.

The convertor can deal with both label-free and labeled example files. The main difference between these files lies in the column “IsotopeLabelType” – for label-free files the value will always be “light,” for files derived from a labeled-based study, the value could be ‘light’ or “heavy.” Additionally, there is no <*RatioList*> produced for label-free mzQuantML output. The convertor also performs simple summation of quantified features (sum of “XIC area” for each feature of a given peptide) up to modified peptide, populating the results into <*AssayQuantLayer*> under <*PeptideConsensusList*> (Fig. 2). The correct CV terms for describing the experimental design are appended to the <*AnalysisSummary*> element on this basis, as well as parameters being added to the <*DataProcessing*> section to capture a trace of the metadata of the analysis steps performed.

Anubis is a software package aimed for automated analysis of SRM data, with probability estimation for detected peptides and with correction of interfering signals [12]. To further validate the SRM extensions for mzQuantML, and to facilitate its uptake, we added mzQuantML export to the standard Anubis distribution, available at http://quantitativeproteomics.org/anubis. The implementation uses the jmzQuantML library for writing.

As well as the tools reported here for exporting mzQuantML-SRM files, a number of other developments are on-going for importing mzQuantML. Our group and colleagues have developed the mzQuantML viewer and library (http://code.google.com/p/mzq-lib/) [14]. In the viewer, users can open mzQuantML files (including those files containing SRM data) and view different *QuantLayers* in the file. It can also generate line plots for a row of data across assays. The library contains routines for postprocessing mzQuantML files (peptide to protein inference, normalization, statistics, and so on), and has also been updated to process SRM-based mzQuantML files. Lastly, we are in discussions with the developers of the PASSEL public repository, and we will work with the PASSEL team to enable mzQuantML upload, as more tools start to export in this format. The PRIDE database already accepts mzQuantML upload, and while PRIDE is broadly intended for discovery mode LC-MS data, for quantitative data sets that include some aspects of SRM, mzQuantML SRM files provide a potential route for data submission.

The updated specification document for mzQuantML (1.0.1) has been reviewed in parallel by the PSI document process, associated with three example files, two converted from Skyline output and one produced by Anubis. They can be found in http://code.google.com/p/mzquantml/source/browse/#svn/trunk/examples/version1.0/files_under_development/SRM. There is currently almost no quantitative SRM data deposited in public databases in a format that allows for straightforward reprocessing or, for example, assessment of the method used to infer protein-level abundance values from SRM signals. We anticipate that the release of the SRM update for mzQuantML will foster improved data sharing between different tools, will facilitate submissions to public databases and will improve abilities for assessment and potential reprocessing of SRM-based quantitative datasets.

## Abbreviations

CV: control vocabulary
PSI: proteomics standards initiative
